# Bacterial Pathogens and Antimicrobial Resistance Patterns in Pediatric Urinary Tract Infections: A Four-Year Surveillance Study (2009–2012)

**DOI:** 10.1155/2014/126142

**Published:** 2014-05-19

**Authors:** Seyed Reza Mirsoleymani, Morteza Salimi, Masoud Shareghi Brojeni, Masoud Ranjbar, Mojtaba Mehtarpoor

**Affiliations:** ^1^Department of Nursing, Faculty of Nursing and Midwifery, Shahid Beheshti University of Medical Sciences, Tehran 1985717443, Iran; ^2^Department of Physiology, Faculty of Medicine, Shahid Beheshti University of Medical Sciences, Tehran 1985717443, Iran; ^3^Student Research Committee, Hormozgan University of Medical Sciences, Bandar Abbas 7914964153, Iran; ^4^Department of Health Management and Economics, School of Public Health, Tehran University of Medical Sciences, Tehran 1417614411, Iran

## Abstract

The aims of this study were to assess the common bacterial microorganisms causing UTI and their antimicrobial resistance patterns in Bandar Abbas (Southern Iran) during a four-year period. In this retrospective study, samples with a colony count of ≥10^5^ CFU/mL bacteria were considered positive; for these samples, the bacteria were identified, and the profile of antibiotic susceptibility was characterized. From the 19223 samples analyzed, 1513 (7.87%) were positive for bacterial infection. UTI was more frequent in male (54.9%). *E. coli* was reported the most common etiological agent of UTI (65.2%), followed by *Klebsiella* spp. (26%), *Pseudomonas aeruginosa* (3.6%), and *Staphylococcus* coagulase positive (3.7%). Results of antimicrobial susceptibility analysis for *E. coli* to commonly used antibiotics are as follows: Amikacin (79.7%), Ofloxacin (78.3%), Gentamicin (71.6%), Ceftriaxone (41.8), Cefotaxime (41.4%), and Cefixime (27.8%). Empirical antibiotic selection should be based on awareness of the local prevalence of bacterial organisms and antibiotic sensitivities rather than on universal or even national guidelines. In this study, Amikacin and Gentamicin were shown to be the most appropriate antibiotics for empiric therapy of pyelonephritis, but empirical therapy should only be done by specialist physicians in cases where it is necessary while considering sex and age of children.

## 1. Introduction

Urinary tract infection (UTI) is a common health problem during the childhood period and it is an important cause of morbidity and mortality in the first 2 years of life [1–4]. The reported incidence of UTI is 7% among girls and 2% among boys during the first 6 years of life [5]. The main objects in childhood urinary tract infections are rapid recovery from complaints and prevention of related complications, such as urosepsis, urolithiasis, and renal abscess, as well as the prevention of permanent renal parenchymal damage [6]. To achieve these aims, empirical antibiotic prescription is often endorsed even before the culture results are available [7]. On the other hand, antibiotic resistance of urinary tract pathogens has been known to increase worldwide, especially to commonly used antimicrobials [8, 9]. The increasing antibiotic resistance trends are likely to have important clinical implications for the empirical use of antibiotics [10]. For this reason, knowledge of the etiology pathogens of UTIs and their antimicrobial resistance patterns in specific geographical locations may aid clinicians in choosing the appropriate antimicrobial empirical treatment [11]. Prior to this study, the frequency of bacterial species causing UTI and their susceptibility patterns to most commonly used antibiotics has not been previously determined in the southern provinces of Iran; so the aim of this study was to characterize these factors in this region of Iran.

## 2. Methods

This retrospective study analyzed the bacteria isolated from patients with UTI at the Children's Hospital, the main center for newborns and children located in Bandar Abbas, capital of Hormozgan Province, South of Iran. Urine samples were obtained from outpatients and inpatients with suspected UTI and those who were admitted in pediatric wards with signs and symptoms of UTI to document the common bacterial species causing UTI and their antibacterial susceptibility profile. The period of study was from 2009 to 2012. Patients were children aged from 1 week to 16 years without history of genitourinary abnormalities, recent hospitalization, or antibiotic usage. Patients were hospitalized for evaluation and treatment with signs and symptoms of acute pyelonephritis, including: temperature ≥ 38°C, chills, frequency, dysuria, urgency, suprapubic and/or flank tenderness, pyuria (defined as ≥5 WBC/Hpf), and fever with unknown source in children and in neonates (7–30 days of age) with clinical evidences of sepsis.

Data on age, sex, result of urine culture, the etiological agent, and susceptibility pattern were obtained from the medical records of patients. Urine samples were collected using midstream method in toilet-trained children and using clean-catch methods or sterile bladder catheter in younger children and infants.

Samples were inoculated on blood agar and eosin methylene blue agar plates and then were read after overnight incubation at 37°C. After incubation, the urine culture samples were classified as negative, positive, and contaminated. When polymorphic bacterial growth (two or more bacterial species growth in one plate) was observed, the samples were classified as contaminated (exclusion criteria). The urine cultures were considered as negative when bacterial growth was lower than 10^3^ CFU/mL (exclusion criteria). Growth of two or more bacterial species (polymorphic bacterial growth) was considered as an exclusion criterion. When monomorphic bacterial growth was higher than 10^5^ CFU/mL, the culture was classified as positive (inclusion criteria) and, for these cases, the antimicrobial susceptibility test (AST) was performed. The AST was also performed when the result of urine culture was between 10^4^ and 10^5^ CFU/mL. Identification of bacterial microorganisms was made on the basis of gram reaction, morphology and biochemical features. The AST was performed by Kirby-Bauer disk diffusion method. A bacterial suspension in physiological saline solution was prepared by picking up 1-2 colonies from pure cultures. The suspension was spread on Mueller-Hinton Agar plate by a swab. Antibiotic disks were placed onto the cultures medium surface. The culture plates were incubated at 37°C for 24 hours; then inhibition zones were measured and hereby the antimicrobial efficacy was determined [12]. The commercial antibiotics used for isolates included Ciprofloxacin, Trimethoprim-sulfamethoxazole (Cotrimoxazole), Gentamicin, Tobramycin, Ampicillin, Nitrofurantoin, Nalidixic acid, Ceftriaxone, Cefotaxime, Cefalexin, Cefazolin, Amoxicillin, Oxacillin, Cefixime, Ceftazidime, Erythromycin, Tetracycline, Clindamycin, Ofloxacin, and Amikacin; in addition to these antibiotics, Penicillin and Erythromycin were used for gram positive bacteria. The data were analyzed using the Statistical Package for the Social Sciences (SPSS) 16.0 for Windows. The normality of data and homogeneity of variance were checked before analysis. As most of the variables failed, these statistical method assumptions and the nonparametric Kruskal-Wallis test, as well as chi square test, were used.

## 3. Results

From January 2009 to 2012, a total of 19223 urine samples were submitted for analysis and culture. These samples showed 1513 (7.87%; 95% CI 7.49–8.2%) bacterial growth higher than 10^5^ CFU/mL. The prevalence of UTI among male and female children suspected to have UTI was 5.1% (845/16282; 95% CI 4.78–5.43%) and 22.4% (659/2941; 95% CI 20.9–23.9%), respectively. Only the first urine sample of one patient and monomorphic bacterial growth samples was considered in this study. 176 of these samples were* Staphylococcus *coagulase negative (rather a contamination microorganism) and were excluded from the study. So, finally, 1209 samples were included. 664 (54.9%) of these patients were males and 545 (45.1%) were females. Of these patients, 437 (36.1%), 377 (31.2%), and 395 (32.7%) were neonates (<28 days), infants (28 days to 1 years), and children (1 years to 14 year), respectively. The predominant agents of UTI were successively* E. coli* (65.2%; 95% CI 62.5–67.8%),* Klebsiella *spp. (26%; 95% CI 23.6–28.4%),* Pseudomonas aeruginosa* (3.6%; 95% CI 2.6–4.6%),* Staphylococcus* coagulase positive (3.7%; 95% CI 2.7–4.7%),* Citrobacter* (0.9%; 95% CI 0.4–1.3%),* Enterobacter* spp. (0.4%; 95% CI 0.1–0.7%), and* Proteus mirabilis* (0.2%; 95% CI 0.0–0.4%) (Table 1).

Analysis of the results according to patient gender represented that, although* E. coli* is the predominant isolated pathogen from both sexes, it occurred more frequently in females (70.8% in females compared to 60.5% in males: significant at *ρ* = 0.001 (chi square = 11.359)), whereas the prevalence of UTI due to* Klebsiella* spp. was higher in males than in females (28.3% in males compared to 23.1% in females, respectively. Significant at *ρ* = 0.001 (chi square = 11.359)). And the prevalence of UTI caused by* Pseudomonas aeruginosa* was significantly higher in males at *ρ* = 0.035 (chi square = 4.45).

**Figure 1:**
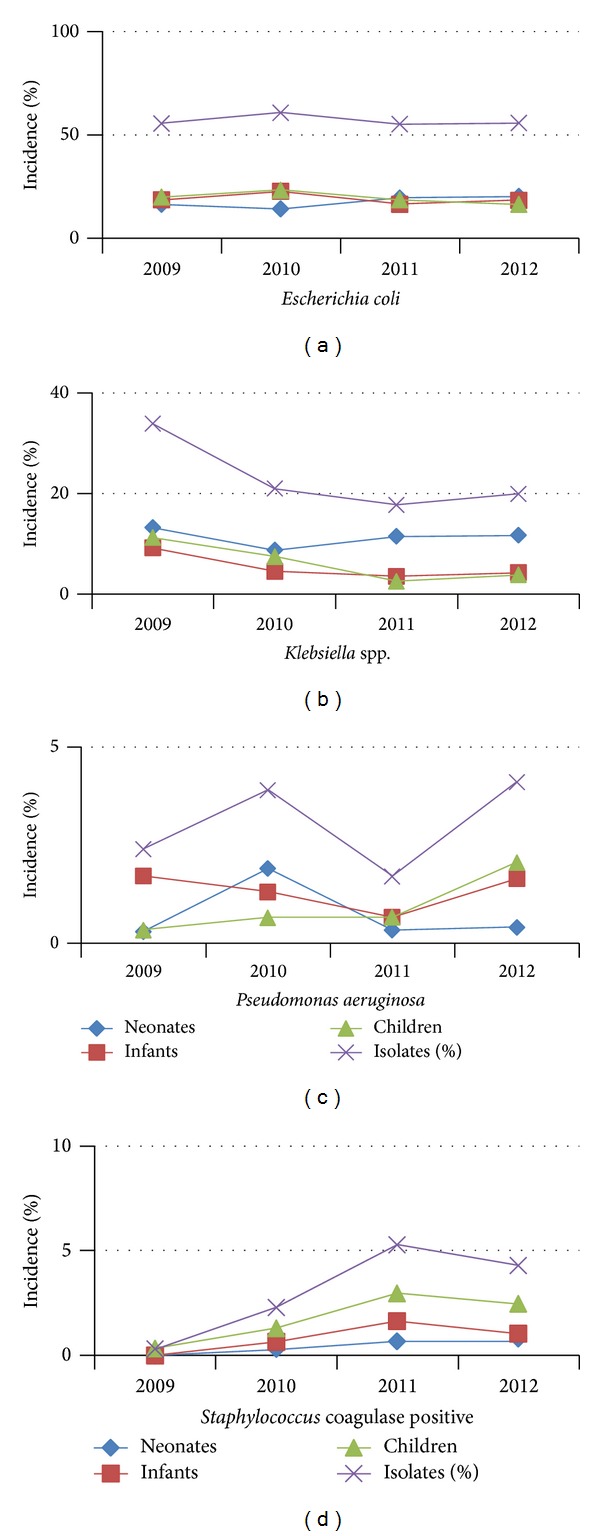
Incidence of the main bacteria implicated in UTI by age during the study period.

Table 1 illustrates incidence of the main bacterial pathogens implicated in urinary tract infection, according sex and age during the study period. Significant (Kruskal-Wallis test, *ρ* < 0.05) changes in the main bacterial pathogens responsible for UTI were observed during the study period. In general, the incidence of* Staphylococcus* coagulase positive increased and the incidence of* Klebsiella* spp. decreased during the period of the study (Figure 1).

**Figure 2:**
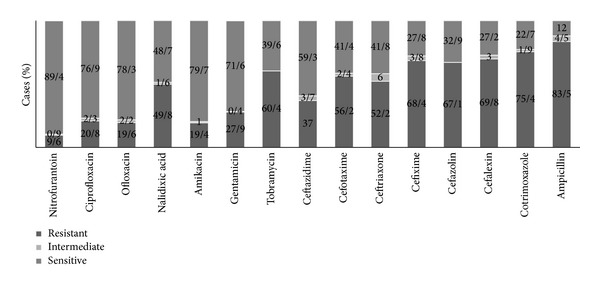
*E. coli* sensitivity and resistance pattern of bacteria causing UTI to antibiotics.

**Figure 3:**
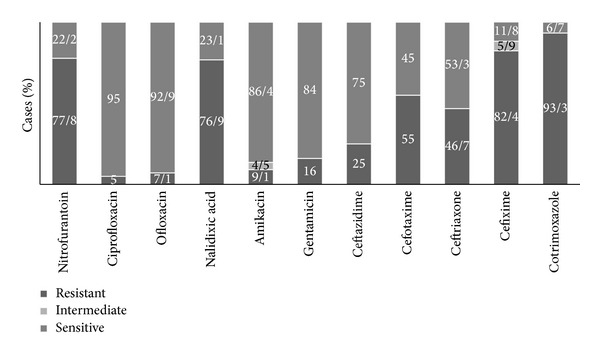
*Pseudomonas aeruginosa *sensitivity and resistance pattern of bacteria causing UTI to antibiotics.

*In vitro* sensitivity testing showed that the mean susceptibility* E. coli* had a sensitivity rate of 79.7% to Amikacin and 77.1% to Ciprofloxacin. In this study, the highest resistance rate of this germ was to Ampicillin (83.5%) followed by Trimethoprim-sulfamethoxazole (75.4%). The* E. coli *resistance to Trimethoprim-sulfamethoxazole (one of the most important UTI empirical therapy options) changed significantly over the study period (chi square test, *ρ* < 0.001). In general, the resistance rate has been increased in females but has been reduced in males (Table 2).* Klebsiella *spp., the second common germ producing UTI, showed the highest sensitivity to Ciprofloxacin (81.3%) and Amikacin (73.1%) and the highest resistance to Ampicillin (86.3%) and Cephalothin (62.4%).* Pseudomonas* was 100% resistant to Trimethoprim-sulfamethoxazole in this study. It showed the highest sensitivity to Tobramycin (100%), Amikacin (86.4%), and Gentamycin (84%). The antibiotic resistance patterns of* E. coli* (the most common germ) and* Pseudomonas aeruginosa* (the most antibiotic resistant germ) agents of UTI are presented in Figures 2 and 3.

## 4. Discussion

Urinary tract infection (UTI) is of major clinical importance owing to considerably high morbidity and mortality rates among children [3]. In this study, of 19223 patients who were suspected to UTI and from whom urine samples were taken, only 7.87% had a urinary tract infection. Its relative ratio varies in different areas of Iran. In studies carried out in Tabriz and Qazvin, 13.2% and 7.2% of pediatric suspected of urinary tract infection had a positive urine culture [10, 13] that was similar to our results. This is possibly because UTI symptoms are not a dependable indicator of infection and in children younger than 2 years of age are nonspecific [10] therefore, urine culture of suspected children is necessary for a definitive diagnosis of UTI.

In this study,* E. coli*, as the most common pathogen, incidence among females was significantly higher than in males and incidence of* Klebsiella*, as the second most common pathogen, was significantly higher in males. On the other hand, in this study, 70.5% of UTI caused by* Pseudomonas aeruginosa* were in males and only 29.5% were in females.* Pseudomonas aeruginosa* is an opportunistic uropathogen for community-acquired UTI [12] and also totally resistant to first line empirical antibiotics (special importance of this pathogen) [14]. Be male, received recent antibiotic therapy, have a neurogenic bladder and have a history of urinary tract procedures such as catheterization are known as risk factors for UTI caused by* Pseudomonas aeruginosa* [15]. So similar to other literature, sex might influence the etiology of UTI and most be considered in empirical therapy [12].

In this study, among children who have a positive urine culture, 55.9% were males and 44.1% were females. Also, most of the participants were infants and neonates (65.2%) and 34.8% were children. Male children are infected more than girls during the first 3 months of their life but, incidence of UTI among girls who have more than one year old is much more [16] possibly because the structural anomalies incidence in first 3 months among boys is more. Boys were more at risk of wrong diagnosis of UTI than female children too. In this study, the frequencies of positive cultures were 5.1% for males and 22.4% for females (ratio∼1 : 4). Farajnia et al. reported this ratio 1 : 2 [10] and Farrell et al. reported this ratio 1 : 4.1 [17]. The cause of this needs more study.

Chi square test showed that pathogens differed significantly (*ρ* < 0.001) across the three age groups of study.* E. coli* prevalence was higher in infants (63.1%), incidence of* Klebsiella* was higher in neonate (32.9%), and* Pseudomonas aeruginosa* incidence was higher in infants, but the* Staphylococcus* coagulase positive prevalence was higher in children (5.4%). So age might influence the etiology of urinary tract infection as has been shown in the study of Afsharpaiman et al. [16]. Overall, these results indicate that urine culture is necessary for a definitive diagnosis of UTI and that empirical therapy should only be done by specialist physicians in case where it is necessary while considering sex and age of children.

*E. coli* is the leading uropathogen and was isolated from 56.6–84.6% of Iranian children with febrile UTI [16, 18]. The results of this study in this context are in agreement with previous literature findings.* E. coli* was the most causative organism responsible for 65.2% of urinary tract infections. But the resistance pattern of this germ to antibiotics was very different in comparison with other studies. For example Sharifian et al. reported the highest susceptibility percentage of* E. coli* to Ceftriaxone (97.8%) and Cefotaxime (95.2%) in 2006 in Tehran [19]. But, in this study,* E. coli* specimens were 52.2% and 56.2% resistant to Ceftriaxone and Cefotaxime, respectively, probably because the pattern of the sensitivity of microorganisms to antibiotics varies over time and between different geographical locations. There was no study before this study for physicians of this region to estimate the most common pathogen and its resistant pattern in pediatrics, but they empirically consider* E. coli* as the most causative agent and Ceftriaxone and Cefixime as the most appropriate choice for UTI treatment but the results of this study have shown that Ceftriaxone probably cannot be the best choice hereafter. On the other hand, according to the results of this study,* E. coli* specimens were 64.8% resistant to Cefixime and 75.4% resistant to Trimethoprim-sulfamethoxazole. Although Cefixime probably cannot be the best choice, it is better than Trimethoprim-sulfamethoxazole for outpatient treatment option.

In a study conducted in Khartoum, Ali and Osman reported that the mean susceptibility of all isolates was too high to Gentamicin (96%), Ciprofloxacin (94%), and Ceftriaxone (90%) whereas the lowest percentages of susceptibility were reported for Amoxicillin clavulanate (19%) and Ampicillin (14%) [20]. In a study conducted in the US hospitals, resistance among* E. coli* was highest for Trimethoprim-sulfamethoxazole (24%) but lower for Nitrofurantoin (<1%) and Cephalothin (15%) [5]. This study revealed a high* E. coli* resistance rate to antibiotics. Except for Nitrofurantoin and Ciprofloxacin that are not appropriate options for UTI treatment in children [19], the highest percentages of susceptibility were seen for Amikacin (79.7%), Ofloxacin (78.3%), and Gentamicin (71.6%), whereas the highest percentages of resistance for this pathogen were found for Ampicillin (83.5%), Cotrimoxazol (75.4%), and Cefalexin (69.8%). Other studies in Iran have also indicated a high resistance rate to antibiotics. For example, Farajnia et al. reported resistance rate of 90.7% to Ampicillin, 51.8% to Cotrimoxazol, and 26.5% to Cefalexin, whereas the highest percentages of susceptibility were seen for Amikacin (96.6%) and Gentamicin (92.9%) in 2008 in Tabriz [10]. These significantly higher bacterial resistance rates to antibiotics in our country in comparison with other countries seem to be the result of two factors: first, a higher rate of antibiotic usage by families even in the absence of a prescription and, second, a population with a high percentage of young individuals since UTI is more common in the early years of life (UTI is most common in girls aged 3 to 5 years) [19]. The other factor that probably caused this study to show higher bacterial resistant rates of antibiotic is the empirical therapy itself because the results of this study showed that only 7.87%, of who were suspected to UTI, had a urinary tract infection in reality. Probably a high rate of antibiotics using resulted in various antibiotic resistance patterns in different parts of Iran.

In conclusion, we suggest that empirical antibiotic selection should be based on knowledge of the local prevalence of bacterial organisms and antibiotic sensitivities rather than on universal or even national guidelines. In this study, Amikacin and Gentamicin were shown to be the most appropriate antibiotics for empiric therapy of pyelonephritis, but, empirical therapy should only be done by specialist physicians in cases where it is necessary while considering sex and age of children.

## Figures and Tables

**Table 1:** The main bacterial pathogens implicated in urinary tract infection by sex and age throughout the study period.

Bacteria	Neonates			Infants			Children		
	Total^a^ (*N* = 437)	Male^b^ (*n* = 260)	Female^b^ (*n* = 177)	Total^a^ (*N* = 377)	Male^b^ (*n* = 217)	Female^b^ (*n* = 160)	Total^a^ (*N* = 395)	Male^b^ (*n* = 187)	Female^b^ (*n* = 208)
*E. coli *	57.7	54.2	62.7	70.8	66.8	76.3	68.1	62.0	73.6
*Klebsiella *	36.2	38.1	33.3	19.4	21.2	16.8	21	23.0	19.2
*P. aeruginosa *	2.3	3.1	1.1	5.0	6.0	3.8	3.8	5.3	2.4
*S. coagulase positive *	1.6	2.3	0.6	3.2	4.1	1.8	6.6	9.2	4.3
*Citrobacter *	1.6	1.5	1.7	0.8	0.5	1.3	0.3	0.0	0.5
*Enterobacter *	0.6	0.8	0.6	0.5	0.9	0.0	0.0	0.0	0.0
*Proteus *	0.0	0.0	0.0	0.3	0.5	0.0	0.3	0.5	0.0

^a^Percentage determined in relation to *N*; ^b^percentage determined in relation to *n*.

**Table 2:** The *E.  coli* resistance to Trimethoprim-sulfamethoxazole over the study period.

	Year				*P* value	chi square
	2009	2010	2011	2012		
Male	72.7%	81.7%	47.9%	64.0%	<0.001	33.961
Female	68.4%	72.3%	66.7%	86.9%	0.004	19.201

Total	70.6%	77.6%	56.6%	73.5%	<0.001	40.589
